# Rotational stability of monofocal and diffractive multifocal toric intraocular lens with identical design and material: a propensity score based prospective comparative study

**DOI:** 10.1186/s12886-024-03281-4

**Published:** 2024-02-16

**Authors:** Runhan Shi, Dongmei Ma, Qiulin Zeng, Zhixiang Hua, Wenqian Shen, Lei Cai, Jin Yang

**Affiliations:** 1 Department of Ophthalmology, Eye, Ear, Nose, and Throat Hospital, Fudan University, 83 Fenyang Rd, Shanghai, 200031 China; 2 Key NHC Laboratory of Myopia, Laboratory of Myopia, Fudan University, Chinese Academy of Medical Sciences, Shanghai, China; 3 Shanghai Key Laboratory of Visual Impairment and Restoration, Shanghai, China; 4 Zhongshan Hospital, Fudan University, Shanghai, China; 5 Department of Ophthalmology, Shanghai Xinshijie Dongqu Eye Hospital, Shanghai, China

**Keywords:** Propensity-matched analysis, Astigmatism, Monofocal toric IOL, Multifocal toric IOL, Rotation

## Abstract

**Purpose:**

To compare the rotational stability of a monofocal and a diffractive multifocal toric intraocular lens(IOLs) with identical design and material.

**Methods:**

This prospective study enrolled patients who underwent plate-haptic toric IOL (AT TORBI 709 M and AT LISA 909 M) implantation. Propensity score matching (PSM) was performed to balance baseline factors. Follow-up examinations were conducted at 1 h, 1 day, 3 days, 1 week, 2 weeks, 1 month, and 3 months postoperatively. A linear mixed model of repeated measures was used to investigate the changes in IOL rotation over time. A 2-week timeframe was utilized to assess differences in IOL rotation between the two groups.

**Result:**

After PSM, a total of 126 eyes were selected from each group for further analysis. Postoperatively, the time course of IOL rotation change in the two groups remained consistent, with the greatest rotation occurring between 1 h and 1 day postoperatively. At the 2-week postoperative mark, the monofocal toric IOL exhibited a higher degree of rotation compared to the multifocal toric IOL (5.40 ± 7.77° vs. 3.53 ± 3.54°, *P* = 0.015). In lens thickness(LT) ≥ 4.5 mm and white-to-white distance(WTW) ≥ 11.6 mm subgroups, the monofocal toric IOL rotated greater than the multifocal toric IOL (*P* = 0.026 and *P* = 0.011, respectively).

**Conclusion:**

The diffractive multifocal toric IOL exhibits superior rotational stability compared to the monofocal toric IOL, especially in subgroups LT ≥ 4.5 mm and WTW ≥ 11.6 mm. Moreover, the time course of IOL rotation change is consistent for both, with the maximum rotation occurring between 1 h and 1 day postoperatively.

## Introduction

Approximately 30% of cataract surgery patients have preoperative corneal astigmatism of 1.00 diopters (D) or higher [1, 2]. Astigmatism plays a crucial role in determining the postoperative visual acuity recovery and overall improvement in visual quality for these patients. Several techniques have been developed to address preoperative corneal astigmatism during cataract surgery, including toric intraocular lens (IOL) implantation. The toric IOL is an intraocular lens that combines the cylindrical power for correcting astigmatism with the spherical power of the IOL. It can counteract regular corneal astigmatism and significantly reduce the residual astigmatism in patients after cataract surgery, thereby improving the rate of spectacle independence [3–5]. However, the accurate axial positioning of toric IOL is crucial for achieving optimal astigmatism correction and preserving high-quality vision. 1-degree rotation can neutralize the astigmatism correction by 3.3%, and a rotation of 30 degrees can even completely eliminate the correction effect [5, 6].

Research findings indicate that various factors, including ocular biometric parameters, the material and haptic design of the IOL, surgical factors, and other external influences, can impact the postoperative rotational stability of the IOL. The toric AT TORBI 709 IOL (Carl Zeiss Meditec AG) and toric AT LISA 909 M IOL (Carl Zeiss Meditec AG) are two types of toric IOLs with the same material and flat haptic design. However, they differ in terms of the IOL surface [7, 8]. It has been noted in a previous study that the design of diffractive rings on the surface of the C-loop haptic IOL may also influence its rotational stability [9]. However, it remains unclear that whether the diffractive rings on IOL surface affect the rotational stability of the plate-haptic toric IOL. Moreover, the time course of IOL rotation change is of great significance for clinical monitoring. Therefore, this study aimed to compare the rotational stability and the time courses of IOL rotation changes of the two plate-haptic toric IOLs.

## Patients and methods

### Study subjects

This prospective observational study included cataract patients who received outpatient cataract surgery and the implantation of either toric AT TORBI 709 M IOL or toric AT LISA 909 M IOL (Carl Zeiss Meditec AG) between March 2021 and December 2022. The study included cataracts patients with regular astigmatism of at least 0.75 D, but excluded those with preexisting irregular astigmatism, corneal disease, small pupil, glaucoma, a history of ocular surgery or trauma, previous uveitis or retinal detachment, retinitis pigmentosa, zonular weakness, or intraoperative complications such as anterior capsuletears, anterior chamber hemorrhage, and posterior capsule rupture. The study was approved by the Institutional Review Board of the Eye and ENT Hospital of Fudan University and followed the Declaration of Helsinki. All patients gave written informed consent.

### Intraocular lens

The toric AT TORBI 709 M IOL is a monofocal aspheric toric IOL, which is made of hydrophobic surface-modified hydrophilic acrylate, with a plate-haptic design and an overall diameter of 11.0 mm, an optic diameter of 6.0 mm, and spherical powers ranging from − 10.0 to + 32.0 D and cylinder powers ranging from + 1.0 to + 12.0 D in increments of 0.5 D. The toric AT LISA 909 M IOL is a multifocal toric IOL with the same material and dimension characteristics as the toric AT TORBI 709 M IOL but unpolished surface (diffractive rings on the anterior surface). Regardless of the pupil size, the IOL has asymmetrical light distribution across 2 foci: 65% for distance and 35% for near vision.

### Preoperative examinations

Prior to surgery, all patients underwent a comprehensive ophthalmic examination that included an assessment of visual acuity, silt-lamp microscopy, intraocular pressure measurement, corneal tomographyusing the Pentacam HR (Oculus Optikgerate GmbH), ocular biometric measurement using the IOLMaster 700 (Carl Zeiss Meditec AG), optical coherence tomography using the Cirrus HD-OCT (Carl Zeiss Meditec AG), B-scan ultrasonography, ultra-wide-angle scanning laser ophthalmoscope (Daytona P200T), and fundoscopy. The power and alignment of the toric IOL were determined using the manufacturer’s online calculator, based on ocular biometric parameters(https://zcalc.meditec.zeiss.com/).

### Surgery

Limbal marks were made at the 0-degree and 180-degree positions while the patients were seated at the slit lamp microscope with their heads vertically aligned. An experienced surgeon (J.Y.) performed all surgical procedures using the Callisto Eye System (Carl Zeiss Meditec AG) as a guide. The surgery involved performing continuous curvilinear capsulorhexis with a 5.4 mm diameter through a 2.3 mm corneal incision, followed by hydrodissection, phacoemulsification, and capsular polishing. The IOL was then carefully implanted into the capsular bag and rotated to the desired position. After removing the viscosurgical material and verifying the IOL axis, the incisions were hydrated. Patients were required to rest near the operating room during the first hour after surgery, and were prescribed prednisolone (1%) and levofloxacin (0.5%) for 2 weeks and pranoprofen (0.1%) for 4 weeks following surgery.

### Postoperative examination

All patients were followed at 1 h, 1 day, 3 days, 1 week, 2 weeks, 1 month, and 3 months, postoperatively, and received comprehensive ophthalmic evaluation, which included assessments of visual acuity, refraction, and slit-lamp microscopy at the outpatient clinic. To ensure the proper functioning of the implanted toric IOL, the IOL axis were carefully examined and recorded using a slit-lamp microscope after fully dilating the pupil at each visit. In the event that the IOL rotated more than 10 degrees and exhibited a remaining positive cylinder of more than 1 D, repositioning surgery was conducted at the end of the second week postoperatively.

### Propensity score matching

The purpose of propensity score matching is to reduce the influence of confounding variables and ensure a more balanced comparison between treatment groups. In this study, propensity score matching was employed to balance significant baseline characteristics that might interfere with the rotation and other parameters of toric IOLs. Logistic regression analysis was used to estimate the propensity scores, taking into account covariates such as age, axial length (AL), anterior chamber depth (ACD), lens thickness (LT), white-to-white distance (WTW), and preexisting corneal astigmatism (ΔK). Subjects in the monofocal toric IOL group were matched 1:1 with those in the multifocal toric IOL group based on the propensity score, using a radius matching algorithm without replacement with a caliper value of 0.02. The standardized mean difference for each included covariate was calculated before and after matching to assess the balance between the matched subjects. Only subjects with an eligible match were included in further analysis.

### Statistical analyses

All statistical analyses were performed using SPSS version 26, while GraphPad Prism 8 was employed for graph creation. A linear mixed model with repeated measures and a block-diagonal covariance structure was utilized to examine the time courses of IOL rotation changes. Bonferroni correction was applied for within-group change over time. The overall IOL rotation two weeks after cataract surgery in the subgroup analysis was evaluated using unpaired t-test. Repeated values obtained from the model were presented as the predicted mean ± standard error, while descriptive data were expressed as the mean ± standard deviation. A significance level of 0.05 (two-tailed) was applied for all statistical tests.

## Result

Among 513 eligible patients, 496 eyes of 496 patients who provided written informed consent were recruited for inclusion in this study; 422 eyes (85.1%) of 422 patients completed at least the 3-month follow-up (178 eyes in the monofocal toric IOL group and 244 eyes in the multifocal toric IOL group). Among these patients, the baseline characteristics, except AL and WTW, were significantly different between the two groups. After developing the propensity score, 126 eyes of 126 patients in the monofocal group were matched with 126 eyes of 126 patients in the multifocal group (Table 1).

**Table 1:** Baseline characteristics

Parameters	Before PSM					After PSM				
	Group A (*n* = 178)		Group B (*n* = 244)		P	Group A (*n* = 126)		Group B (*n* = 126)		P
	Mean	SD	Mean	SD		Mean	SD	Mean	SD	
Age (year)	67.92	13.47	61.65	13.43	0.000*	65.37	13.14	64.50	12.13	0.584
AL (mm)	25.23	2.61	24.88	2.07	0.142	24.96	2.34	24.93	2.13	0.92
WTW (mm)	11.64	0.46	11.67	0.46	0.440	11.60	0.48	11.65	0.46	0.392
LT (mm)	4.46	0.49	4.35	0.51	0.022*	4.39	0.50	4.41	0.49	0.834
ACD (mm)	3.13	0.48	3.23	0.44	0.024*	3.14	0.49	3.18	0.42	0.453
ΔK (D)	2.06	0.90	1.74	0.67	0.000*	1.80	0.62	1.91	0.75	0.19

PSM = Propensity score-matched; AL = axial length; WTW = white-to-white diameter; LT = lens thickness; ACD = anterior chamber depth; ΔK = preexisting corneal astigmatism; D = diopter. Group A: eyes that implanted with the monofocal toric IOL group; Group B: eyes that implanted with the multifocal toric IOL group

* Statistically significant (*P* < 0.05)

### The time course of absolute IOL rotation change

**Fig. 1:**
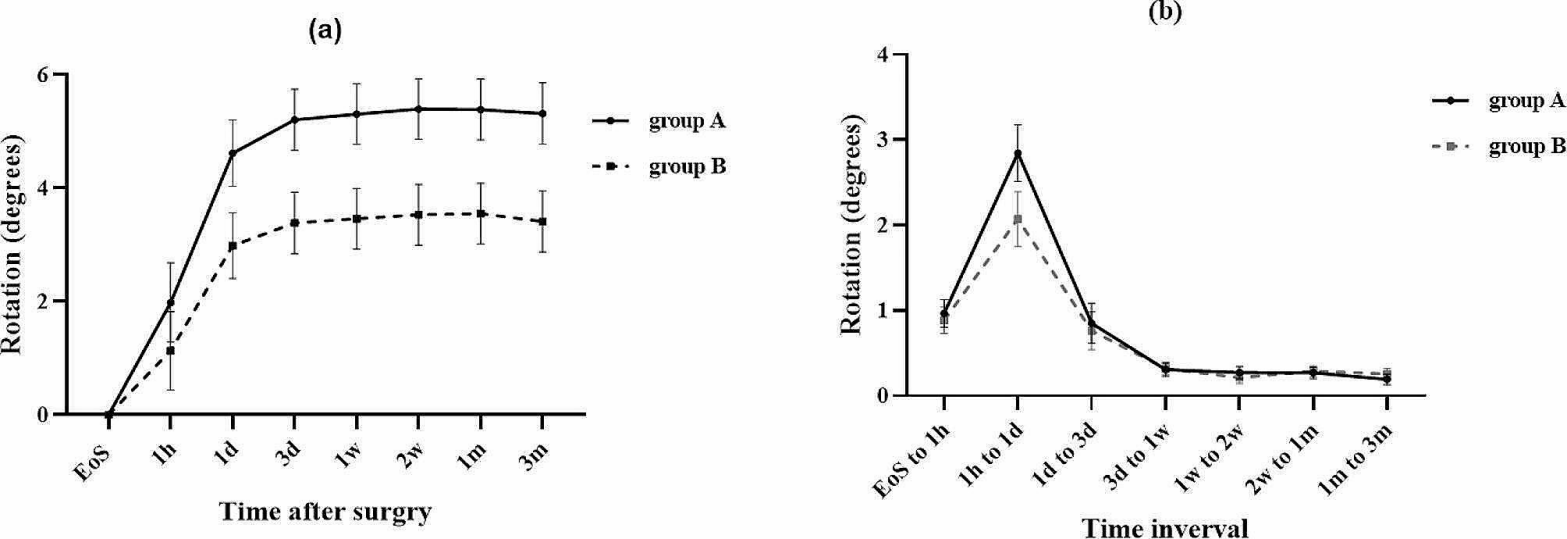
The absolute rotation of toric IOL of both groups. (**a**) The absolute toric IOL rotation of both groups at the three-month follow-up (Mean ± SE). 1.981 ± 0.701 degrees vs. 1.132 ± 0.692 degrees at 1 h, 4.617 ± 0.583 degrees vs. 2.986 ± 0.580 degrees at 1 day, 5.206 ± 3.387 degrees vs. 3.387 ± 0.538 degrees at 3 days, 5.307 ± 0.537 degrees vs. 3.460 ± 0.537 degrees at 1 week, 5.397 ± 0.537 degrees vs. 3.532 ± 0.537 degrees at 2 weeks, 5.388 ± 0.537 degrees vs. 3.552 ± 0.537 degrees at 1 month, and 5.317 ± 0.540 degrees vs. 3.413 ± 0.539 degrees at 3 months, *P* = 0.582. (**b**) Changes in the absolute toric IOL rotation of both groups at different time intervals (Mean ± SE). 0.964 ± 0.160 degrees vs. 0.885 ± 0.153 degrees from the end of surgery to 1 h, 2.845 ± 0.332 degrees vs. 2.072 ± 0.320 degrees from the end of surgery to 1 h, 0.848 ± 0.232 degrees vs. 0.762 ± 0.225 degrees from 1 day to 3 days, 0.305 ± 0.077 degrees vs. 0.318 ± 0.075 degrees from 3 days to 1 week, 0.271 ± 0.069 degrees vs. 0.213 ± 0.068 degrees from 1 week to 2 weeks, 0.269 ± 0.069 degrees vs. 0.287 ± 0.066 degrees from 2 weeks to 1 month, 0.190 ± 0.065 degrees vs. 0.254 ± 0.063 degrees from 1 month to 3 months, respectively, all *P* < 0.05. Group A: eyes that implanted with the monofocal toric IOL group; Group B: eyes that implanted with the multifocal toric IOL group. EOS = End-of-surgery

Figure 1a exhibited the absolute rotation of the toric IOL in relation to the intraoperative positioning offset at multiple postoperative time points, including 1 h, 1 day, 3 days, 1 week, 2 weeks, 1 month, and 3 months. Moreover, the changes in absolute IOL rotation at several time interval was shown in Fig. 1b. No significant group-time interaction was noted when investigating the changes in IOL rotation over time in the two groups, indicating that the time courses of absolute IOL rotation change were identical for the two toric IOLs (*P* = 0.582). Moreover, there was a significant difference in the absolute IOL rotation change at varied time intervals in both groups, with the greatest IOL rotation occurring from 1 h to 1 day after cataract surgery (all *P* < 0.05).

### Overall IOL rotation

Notably, a 2-week timeframe was employed to evaluate intergroup disparities in IOL rotation due to two primary reasons. Firstly, the cumulative absolute rotation across subsequent time points may not holistically capture the comprehensive IOL rotation, considering the potential variations in rotation direction at different intervals. Secondly, the toric IOL demonstrated relative stability two weeks post-cataract surgery, as evidenced by minimal absolute rotation (less than 1 degree) between the 2-week and 1-month mark. Table 2 presents a comparison of the 2-week rotation between the two groups. Furthermore, we examined the influence of IOL placement on postoperative toric IOL stability. Our findings revealed that at 2-week postoperatively, the monofocal group exhibited a higher degree of rotation compared to the multifocal group (5.40 ± 7.77 degrees vs. 3.53 ± 3.54 degrees, *P* = 0.015), particularly in the horizontal direction (5.97 ± 8.44 degrees vs. 3.21 ± 2.82 degrees, *P* = 0.019). Oblique implantations generated more rotation regardless of the IOL(Table 3). The highest recorded rotation was 54°in monofocal group, while in multifocal group, the maximum rotation reached 19°. Additionally, four patients, three from the monofocal group and one from the multifocal group, required surgical realignment.

**Table 2:** Postoperative distribution of toric IOL rotation

IOL rotation (°)	Total N (%)			Clockwise N (%)			Counter-clockwise N (%)	
	Group A	Group B		Group A	Group B		Group A	Group B
≤ 5.0	90 (71.4%)	98 (77.8%)		30 (23.8%)	36 (28.6%)		45 (35.7%)	44 (34.9%)
> 5, ≤ 10	21 (16.7%)	23 (18.3%)		15 (11.9%)	13 (10.3%)		6 (4.8%)	10 (7.9%)
> 10, ≤ 15	6 (4.8%)	3 (2.4%)		1 (16.7%)	0 (0%)		5 (83.3%)	3 (2.4%)
> 15	9 (7.1%)	2 (1.6%)		3 (2.4%)	2 (1.6%)		6 (4.8%)	0 (0%)

**Table 3:** Influence of IOL target axis on postoperative toric IOL rotation

Target axis		Total		Horizontal		vertical		Oblique
Group A		5.40 ± 7.77		5.97 ± 8.44		4.38 ± 6.60		6.29 ± 8.34
Group B		3.53 ± 3.54		3.21 ± 2.82		3.48 ± 3.57		5.89 ± 6.23
*P* Value		0.015*		0.019*		0.369		0.915

* Statistically significant (*P* < 0.05)

### Subgroup analysis

**Fig. 2:**
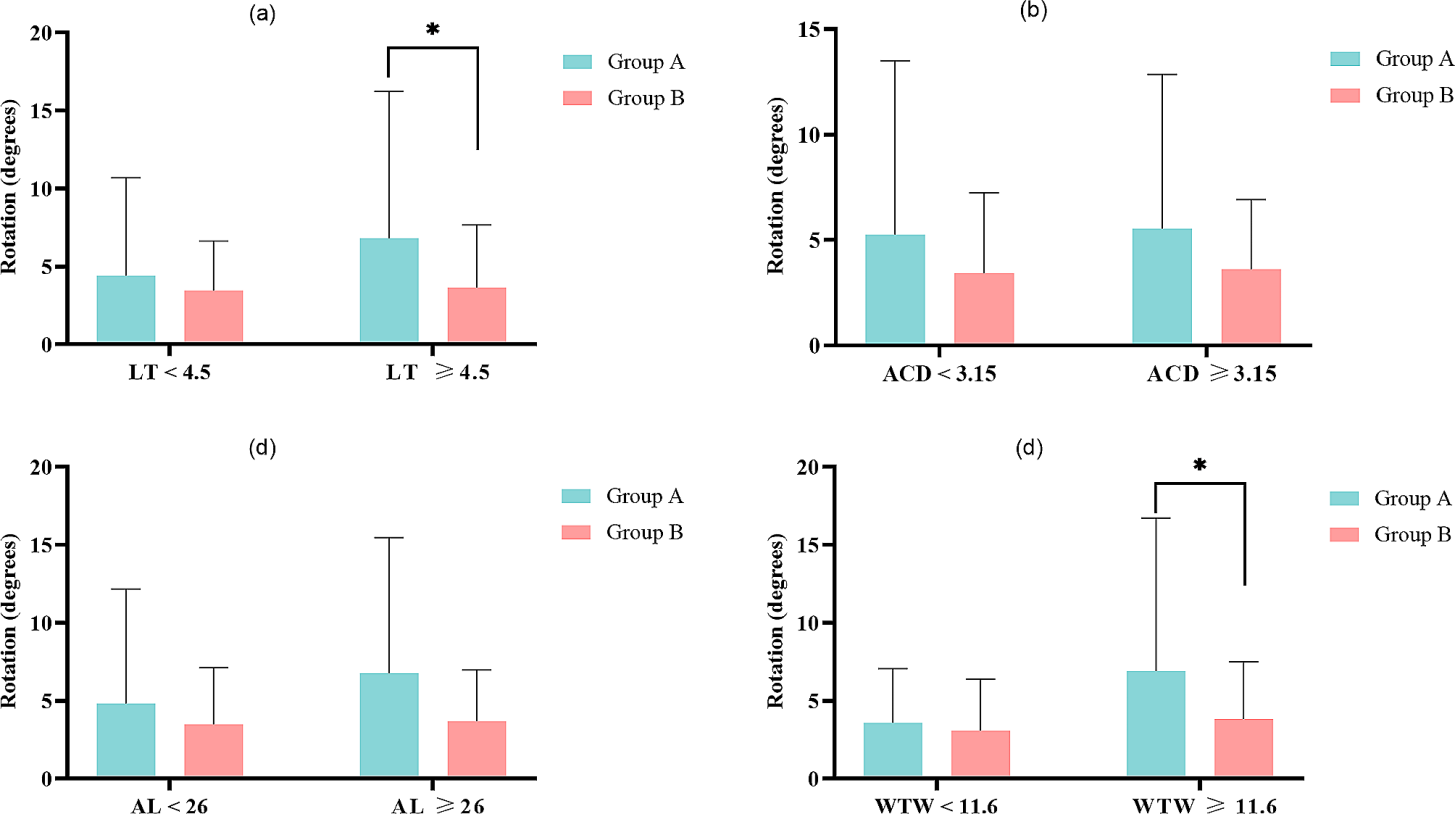
Comparisons of 2-week IOL rotation in subgroups. (**a**) Comparison of toric IOL rotation in the LT subgroups (*P* = 0.248 for LT < 4.5 mm and *P* = 0.026 for LT ≥ 4.5 mm); (**b**) Comparison of toric IOL rotation in the ACD subgroups (*P* = 0.119 for ACD < 3.15 mm and *P* = 0.026 for ACD ≥ 3.15 mm); (**c**) Comparison of toric IOL rotation in the AL subgroups (*P* = 0.11 for AL < 26 mm and *P* = 0.076 for AL ≥ 26 mm); (**d**) Comparison of toric IOL rotation in the WTW subgroups (*P* = 0.453 for WTW < 11.6 mm and *P* = 0.011 for WTW ≥ 11.6 mm). Values are expressed as mean ± SD. * Statistically significant (*P* < 0.05)

Building upon our previous study [8, 10], we classified patients based on AL ≥ 26 mm, LT ≥ 4.5 mm, and WTW ≥ 11.6 mm, and the median value of ACD (3.15 mm). The relationship between these parameters and IOL rotation stability was analyzed (Fig. 2). The results showed that in groups with LT ≥ 4.5 mm and WTW ≥ 11.6 mm, the monofocal group exhibited significantly poorer rotation stability compared to the multifocal group (*P* = 0.026 and *P* = 0.011, respectively).

## Discussion

Previous studies have individually explored the rotational stability and visual quality of the toric AT TORBI 709 M or toric AT LISA 909M [7, 8, 11, 12]. However, no research has been conducted comparing the rotational stability of these two plate-haptic toric IOLs with the same design and material but different surfaces. To address this gap, our study is the first to compare the rotational stability of such plate-haptic toric IOLs, represented by toric AT TORBI 709 M and toric AT LISA 909 M, and to explore the impact of implantation axis and biometric parameters of the anterior segment on rotational stability. It is worth noting that we employed the propensity score matching method to simulate real-world conditions, enhancing the credibility and reliability of our research findings regarding the superior rotational stability of diffractive multifocal toric IOL.

Our study involved a follow-up assessment of the rotational stability of toric IOL at multiple time points within a three-month period. We observed no significant time course of the rotational stability between the groups during the three months after the surgery. The rotational behavior over time was consistent for both types of toric IOLs. In addition, we found that the maximum amount of rotation for both types of toric IOLs occurred within the first hour to one day after the surgery. Previous studies have reported inconsistent results regarding the timing of maximum rotation for toric IOLs, with certain studies suggesting that the maximum rotation occurs within the first hour after the surgery [13, 14]. This discrepancy may be due to the fact that patients were instructed to remain in an immobilization position for one hour after the surgery to prevent early postoperative rotation. After that, patients were allowed to leave the hospital until their scheduled follow-up visit on the next day.

In terms of overall rotation, we compared the two types of toric IOLs at the two-week mark. The rotational stability of the multifocal toric IOL was found to be superior to that of the monofocal toric IOL. Furthermore, we investigated the impact of implantation axis on the rotational stability of the two types of toric IOLs and found that in the oblique direction, both types of lenses were more prone to rotation. We recommend that patients at higher risk of such rotation consider the implantation of a CTR, minimize movement in the early postoperative period, or (and) undergo more frequent monitoring of IOL positional changes. In the horizontal direction, the degree of rotation of the multifocal toric IOL was significantly smaller than that of the monofocal toric IOL. This could be attributed to the wider horizontal positioning of the capsular bag compared to the vertical position, allowing the lens to rotate more easily in the horizontal direction. Additionally, the surface design of the multifocal IOL increased the friction between the IOL and the capsular bag, thereby enhancing rotational stability.

According to previous studies, there exists a relationship between the postoperative rotational stability of toric IOL and certain design elements, such as the diameter of the IOL, the shape of the IOL and haptics, as well as the materials employed in their construction [9, 15–17]. Previous research has indicated a close association between the rotational stability of toric IOL and the magnitude of friction between the IOL and the equatorial region of the capsular bag [18]. It has been proposed that enhancing the frictional force between the toric IOL and the capsular bag through improvements in the IOL’s surface design can lead to enhanced rotational stability [18]. In this study, the diffractive multifocal toric IOL has exhibited superior rotational stability and less total misalignment compared to the monofocal toric IOL. These findings were consistent with a study of 71 eyes conducted by Kristof Vandekerckhove, which compared the FineVision Pod FT trifocal toric IOL to the Ankoris monofocal toric IOL. Vandekerckhove reported that the IOL with an unpolished surface exhibited superior rotational stability, likely attributable to the higher frictional coefficient associated with its surface characteristics [9]. We hypothesize that the better rotational stability observed in the multifocal toric IOL group, compared to the monofocal toric IOL group, may be attributed to the diffractive ring design present on the surface of multifocal toric IOL. The diffractive ring introduces additional surface texture and enhance the frictional interaction between the lens haptics and the capsular bag. This increased friction effectively counteracts a portion of the rotational torque exerted on the IOL, thereby contributing to enhanced rotational stability. This adhesive property plays a crucial role in facilitating the rotational stability of toric IOL, particularly during the early postoperative period before capsular bag shrinkage takes place.

Previous investigations have demonstrated that ocular biological parameters, such as lens thickness (LT), anterior chamber depth (ACD), axial length (AL), and white-to-white distance (WTW), exhibit a negative correlation with the degree of postoperative rotation in toric IOL [10, 19, 20]. In our study, we conducted subgroup analysis focusing on these parameters to assess their impact on rotational stability. The results revealed a significantly greater degree of IOL rotation in the monofocal toric IOL group compared to the multifocal toric IOL group within the LT ≥ 4.5 mm and WTW ≥ 11.6 mm subgroups. The WTW reflects the size of the capsular bag, and LT represents the anterior-posterior diameter of the capsular bag [21]. Larger capsular bags result in reduced frictional resistance between the toric IOL and the bag, which is detrimental to the rotational stability of the toric IOL. The diffractive ring design of the multifocal toric IOL increases this frictional resistance. Based on these findings, our study suggests considering the implantation of multifocal toric IOLs for patients exhibiting these specific ocular characteristics, taking into account additional factors beyond rotational stability. Further evaluation of the benefits and considerations associated with both multifocal and monofocal toric IOLs is warranted in relation to the observed ocular characteristics.

This study has certain limitations that need to be acknowledged. Firstly, it is a single-center study focusing on a specific type of intraocular lens, which may limit its applicability and generalizability. Secondly, an ideal study design would involve a randomized controlled trial. To address this, propensity score matching analysis was conducted to mitigate the lack of random assignment and potential bias. Despite these limitations, our investigation demonstrates a clear and genuine difference in rotational stability and time course of IOL rotation change between the two types of toric IOLs examined. To further substantiate the findings presented in this study, further large-scale clinical research is warranted.

## Conclusion

This prospective study demonstrated that within the study population, the multifocal toric IOL exhibited superior rotational stability compared to the monofocal toric IOL. This superiority was particularly evident in subgroups characterized by LT ≥ 4.5 mm and WTW ≥ 11.6 mm. Furthermore, the time course of IOL rotation change was consistent for both types of toric IOLs, with the maximum rotation occurring between 1 h and 1 day after surgery.

## Data Availability

All the data are provided in the article and the supplementary data.
